# COVID-19 and abnormal uterine bleeding: potential associations and mechanisms

**DOI:** 10.1042/CS20220280

**Published:** 2024-02-19

**Authors:** Jacqueline A. Maybin, Marianne Watters, Bethan Rowley, Catherine A. Walker, Gemma C. Sharp, Alexandra Alvergne

**Affiliations:** 1 Centre for Reproductive Health, Institute for Regeneration and Repair, University of Edinburgh, Edinburgh, U.K.; 2 School of Psychology, University of Exeter, U.K.; 3 ISEM, Univ Montpellier, CNRS, IRD, Montpellier, France; 4 School of Anthropology and Museum Ethnography, Oxford, U.K.

**Keywords:** COVID, Endometrial, Endometrium, Menses, Menstruation, Uterus

## Abstract

The impact of COVID-19 on menstruation has received a high level of public and media interest. Despite this, uncertainty exists about the advice that women and people who menstruate should receive in relation to the expected impact of SARS-CoV-2 infection, long COVID or COVID-19 vaccination on menstruation. Furthermore, the mechanisms leading to these reported menstrual changes are poorly understood. This review evaluates the published literature on COVID-19 and its impact on menstrual bleeding, discussing the strengths and limitations of these studies. We present evidence consistent with SARS-CoV-2 infection and long COVID having an association with changes in menstrual bleeding parameters and that the impact of COVID vaccination on menstruation appears less significant. An overview of menstrual physiology and known causes of abnormal uterine bleeding (AUB) is provided before discussing potential mechanisms which may underpin the menstrual disturbance reported with COVID-19, highlighting areas for future scientific study. Finally, consideration is given to the effect that menstruation may have on COVID-19, including the impact of the ovarian sex hormones on acute COVID-19 severity and susceptibility and reported variation in long COVID symptoms across the menstrual cycle. Understanding the current evidence and addressing gaps in our knowledge in this area are essential to inform public health policy, direct the treatment of menstrual disturbance and facilitate development of new therapies, which may reduce the severity of COVID-19 and improve quality of life for those experiencing long COVID.

## Introduction

In recent years, there has been substantial media attention and public concern regarding menstrual disturbance during the COVID-19 pandemic. This may be due to COVID-19 vaccination, infection with the SARS-CoV-2 virus, pandemic-related stress and/or lifestyle changes, yet the independent contribution of each factor to the menstrual cycle is just beginning to be delineated [1–3]. This limits our ability to provide evidence-based advice to women and those who menstruate about the impact of COVID-19 on menstruation [4]. For many women, menstruation is an outward sign of fertility and health, and menstrual disturbances due to COVID-19 may provoke anxiety and stress as well as concern about the perceived risks of immunisation [5]. Further, the menstrual cycle and ovarian sex hormones have the potential to modulate disease susceptibility and severity [6,7], but our understanding of how the menstrual cycle impacts on SARS-CoV-2 infection and longer-term symptoms of COVID-19 also remains limited. This limited understanding of the dual relationship between COVID-19 and menstruation contrasts with the increasing awareness amongst healthcare professionals that the menstrual cycle can be used as a vital sign of female health [8,9] and the greater appreciation among scientists that sex should be considered in immunological studies [10], emphasising the importance of identifying associations and delineating mechanisms in this area.

In this review, we focus on the impact of COVID-19 on menstrual bleeding, rather than its effects on fertility, dysmenorrhoea or other menstrual symptoms which have been the subject of previous reviews [7,11,12]. First, we define typical and abnormal menstrual parameters. Second, we examine the current evidence for and against an association between either SARS-CoV-2 infection, COVID-19 vaccination, longer-term symptoms of COVID-19 or psychological aspects of the COVID pandemic and menstrual disturbance. Third, potential mechanisms causing such associations are discussed, examining our knowledge of how COVID-19 impacts the hypothalamic–pituitary–ovarian (HPO) axis and the consequent effects on endometrial function as well as examination of potential local endometrial dysfunction. We draw on what is known from the study of SARS-CoV-2 pathophysiology in other systems. For example, SARS-CoV-2 uses the angiotensin-converting enzyme 2 (ACE2) and the cell protease type II transmembrane serine protease (TMPRSS2) to bind to the cell and for virus–cell fusion in lung tissue [13,14] and we discuss the potential role in reproductive tissue. Lastly, we review associations between the menstrual cycle and symptoms and outcomes of COVID-19, discussing potential mechanisms. Throughout, sources of bias within existing studies are evaluated before signposting recommendations for future research in this area. We acknowledge that not all women menstruate and that some people who do not identify as women do. Therefore, we use a combination of ‘women’ and ‘people who menstruate’ throughout this review.

## Menstruation and abnormal uterine bleeding

**Figure 1:**
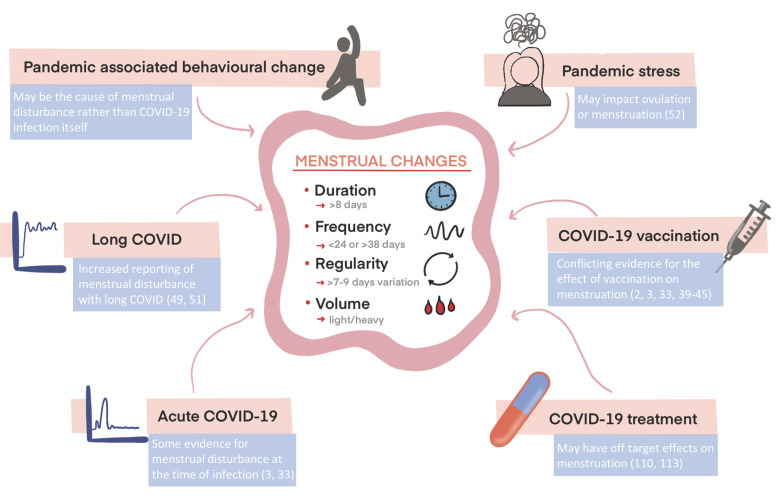
Possible associations between COVID-19 and menstrual disturbance. Summary of the symptoms of abnormal uterine bleeding (AUB), as defined by the FIGO AUB System 1 [15], and the different aspects of COVID-19 which may be associated with reported menstrual changes.

Normal or typical menstrual parameters have been defined by the International Federation of Gynecology and Obstetrics (FIGO) Abnormal Uterine Bleeding (AUB) System 1. Menstrual bleeding typically has a frequency of every 24–38 days, duration of no more than 8 days, variation in shortest to longest menstrual cycle of less than or equal to 7–9 days (age dependant) and a flow volume of subjectively normal [15] (Figure 1). The symptoms of abnormal uterine bleeding (AUB), affecting 1 in 4 women in society [16,17], are debilitating and can result from frequent/infrequent, prolonged, irregular or heavy menstrual bleeding [15].

The symptom of heavy menstrual bleeding (HMB) is defined as excessive menstrual blood loss that interferes with a woman’s physical, emotional, social and/or material quality of life [18]. Depending on the population studied and definition adopted, global figures for HMB vary but are consistently high [19,20]. One in three women have been reported to find their menstrual loss excessive, with this figure rising to one in two as the menopause approaches [21]. Prior to the COVID-19 pandemic, over 800,000 women sought treatment for HMB per year in the UK alone [18] with many more suffering in silence [19]. Assessment of the economic impact of menstrual complaints in the United States (US) prior to the COVID pandemic demonstrated financial losses of greater than $2000 per patient per year due to absence from work and home management costs [22]. Therefore, any additional impact of COVID-19 on menstrual symptoms must be quantified to allow sufficient allocation of resources to address clinical needs.

**Figure 2:**
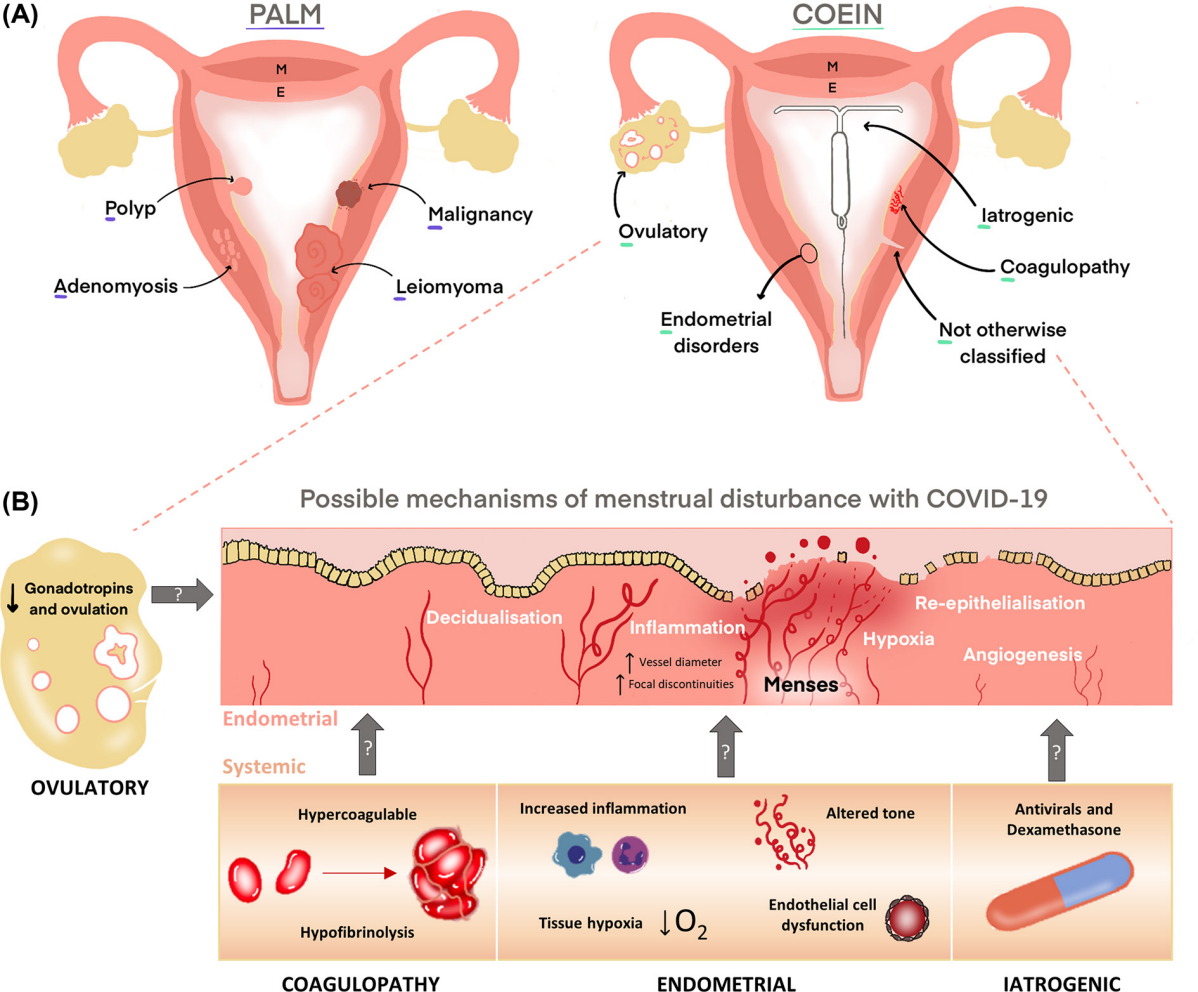
Classification of abnormal uterine bleeding (AUB) using PALM-COEIN, and the hypothesised mechanisms of menstrual disturbance with COVID-19. (**A**) Structural causes of AUB including Polyp, Adenomyosis, Leiomyoma and Malignancy (PALM). Non-structural causes of AUB including Coagulopathy, Ovulatory, Endometrial disorders, Iatrogenic or Not otherwise classified (COEIN) as defined by the FIGO AUB System 2 [15]. (**B**) The hypothesised mechanisms of menstrual disturbance based on reported systemic COVID-19 effects. Focusing on COEI, these effects may impact the endometrial processes required for normal menstruation and repair.

The underlying cause(s) of AUB may be classified as structural or non-structural, as outlined in the International Federation of Gynecology and Obstetrics (FIGO) AUB System 2 [15]. Structural causes are those that can usually be detected during routine examination or investigations (e.g., imaging or histopathology) and include Polyps, Adenomyosis, Leiomyomas (fibroids) or Malignancy (PALM) (Figure 2A). Non-structural causes are not detected on imaging but diagnosed by a detailed clinical history and examination, sometimes supported by laboratory tests. These non-structural causes include coagulopathies, ovulatory disorders, primary endometrial disorders, iatrogenic causes and those that are not otherwise classified (COEIN) (Figure 2A). Identifying the mechanisms underlying menstrual disturbance during COVID is crucial for effective, evidence-based management to maintain menstrual health.

## The association between acute COVID-19 and AUB

There is now increasing evidence that SARS-CoV-2 infection may be associated with menstrual disturbance (Figure 1). Prior to the COVID-19 pandemic, there were few reports of menstrual disturbance associated with infectious disease. Two case reports describe heavy menstrual bleeding as the presenting symptom of Dengue fever [23,24] and genital tuberculosis commonly presents with menstrual disturbance [25]. Early in the pandemic, research on the associations between SARS-CoV-2 infection and menstrual cycle changes was scarce and inconsistent [26]. A retrospective, cross-sectional study was conducted in China in 2020 and included a review of menstrual symptoms in 177 patients admitted to hospital with COVID-19 and 91 non-ovarian infertility patient controls [27]. COVID-19 patients reported more changes in menstrual blood volume (control versus COVID-19, 5% versus 25%, *P*<0.001) and cycle length (control versus COVID-19, 6% versus 28%, *P*<0.001). Review of blood samples from the early follicular phase revealed the average ovarian sex hormone and anti-Müllerian hormone (AMH) concentrations of women of child-bearing age with COVID-19 were similar to those of age-matched controls. However, the external validity of this study has been questioned as the sample is biased towards women with multisystem dysfunction [28]. In addition, it is unclear if controls were hospitalised, which may introduce a significant confounder. Recruitment of 78 female COVID-19 positive patients not using exogenous hormones in Wuhan, China in 2020 revealed higher rates of menstrual disturbance in severe COVID-19 cases versus non-severe cases. None of the changes, however, were significant when comparing the previous three months from recruitment and the initial phase of COVID-19 infection [29]. In the USA, the Arizona CoVHORT study was set up in May 2020 to recruit individuals for a prospective, population-based cohort with the purpose of identifying the long-term consequences of COVID-19 [30]. In a sub-sample of 127 non-pregnant female SARS-CoV-2 positive participants aged 18–45 years, 16% reported changes in their menstrual cycle, including irregular menstruation (60%) and infrequent menstruation (35%) [31]. Yet causality cannot be inferred in this study due to the absence of a control group. In contrast, an association between SARS-CoV-2 infection and menstrual cycle changes was not observed in the Nurses’ Health Study 3, a prospective study of 3,858 pre-menopausal health professionals [2]. In this sample, the prevalence of SARS-CoV-2 infection was low (*n*=421, 11%) compared with vaccination (*n*=3,527, 91%) and more than half of COVID-19 positive individuals (*n*=223) were vaccinated prior to infection, which may have limited the ability of the study to detect small-to-moderate effects. In a study of 182 American women (60% black, 30% Hispanic) recruited in 2019 to 2021 who were not using exogenous hormones, detectable SARS-CoV-2 IgG antibodies (*n* = 36 out of 73 tested) were associated with a higher percentage of self-reported menstrual irregularities (cycles <26 or >35 days in the 3 months prior to survey) [32]. This suggests that SARS-CoV-2 infection may lead to abnormal menstrual cycle parameters, although this study is small and subject to recall bias. In March 2021, an online survey titled ‘The COVID-19 Pandemic and Women’s Reproductive Health’ was conducted in the UK [33]. Menstruating, reproductive aged participants with a history of COVID-19 disease were at an increased relative risk of reporting frequent versus normal cycles (<24 days) (RRR = 1.3 [1.06–1.6], *P*=0.01), heavier versus normal flow (RRR = 1.38 [1.17–1.63], *P*=0.0001) and more versus no changes in mid-cycle spotting (RRR = 1.31 [1.09–1.58], *P*=0.004) and at an increased prevalence of reporting missed periods (PR = 1.31 [1.05–1.64], *P*=0.016) and prolonged period duration (>8 days) (PR = 1.65 [1.08–2.54], *P*=0.02) when compared with those who were vaccinated against COVID-19 but who reported they never had COVID-19. These associations persisted despite adjusting for pre-existing gynaecological conditions and exogenous hormone use. These results suggest that SARS-CoV-2 infection can, in some cases, lead to abnormal cycle parameters, as defined by FIGO AUB System 1 [15]. This survey, carried out as COVID-19 vaccination was being rolled out in the UK and prior to widespread media attention to the impact of vaccination on menstruation, provided the opportunity to separate out vaccine and SARS-CoV-2 infection effects and limited selection bias, but the retrospective methodology remains subject to recall bias and causation cannot be inferred. Most recently, a retrospective cohort analysis of menstrual cycle data collected prospectively by 6,514 users of the period tracker application Clue (https://helloclue.com) examined cycle length in the three cycles prior to COVID-19 symptoms and compared this to the COVID cycle [3]. COVID-19 cycles resulted in a 1.45 day unadjusted increase in cycle length when compared with the average of the three pre-COVID-19 cycles (95% CI: 0.89–2.02, *P*<0.001). A control group (who had neither vaccine nor disease) experienced a 0.68-day decrease (95% CI: −1.18 to --0.19, *P*=0.007) over a similar timeframe. The changes in the COVID-19 group resolved quickly within the next cycle. Sub analysis of a cohort of individuals vaccinated at least 3 months before the onset of COVID-19 (*n* = 2,335) revealed a 1.02-day unadjusted increase in cycle length from the three pre-vaccination cycles compared with the COVID-19 cycle (95% CI: 0.50–1.54, *P*<0.001), suggesting a protective effect of COVID-19 vaccination on experiencing menstrual disturbance with COVID-19. This study, although prospective and focused on app users with regular cycles who did not use hormonal contraceptives, still suffers from selection and recall biases for the date of COVID-19 symptoms.

Taken together, these studies highlight the need for inclusion of standardised menstrual data in large, prospective studies to limit recall and selection bias and provide appropriate control groups. Few of the studies described use standardised nomenclature of menstrual symptoms to describe frequency, duration, regularity and flow volume, as recommended by the FIGO AUB System 1 [15] to facilitate scientific and clinical comparison of menstrual data globally. Confounders such as contraceptive use, pre-existing gynaecological conditions and COVID-19 vaccination status must also be considered in the analysis of menstrual data in the context of acute COVID-19. In addition, none of the included studies record details on SARS-CoV-2 variant and it is possible that there may be disparity in reported menstrual bleeding changes between variants. Despite some inconsistencies in the existing data and the fact that the ideal time for collection of menstrual data for COVID-19 has now passed, there is mounting evidence that SARS-CoV-2 infection is associated with menstrual disturbance. It is, therefore, important that causality and mechanisms are established to inform clinical services and management of those experiencing menstrual disturbance after SARS-CoV-2 infection and to determine how long this disturbance may last after infection.

## The association between COVID-19 vaccination and AUB

Before the COVID-19 pandemic, research on the relationship between vaccination and the menstrual cycle had been limited to prophylactic typhoid [34], human papillomavirus (HPV) [35,36] and hepatitis B vaccines [37]. In an analysis of 99 nurses vaccinated against typhoid in 1911–1913, 53% reported menstrual disturbance, while 47% reported no effects on menstruation [34]. The effects reported were variable, including lighter, heavier, more painful, frequent or infrequent menses. All cases were followed up for 6 months, at which point all menstrual disturbance had resolved. Following HPV vaccination, 71,177 Japanese females completed a questionnaire to investigate the onset of 24 symptoms, including menstrual symptoms [35]. No significant increase in any of the symptoms following HPV vaccination was found. However, they did notice a significant increase in hospital attendance for abnormal amount or regularity of menstrual bleeding, but this did not impact school attendance.

More recently, reports of menstrual disturbances following COVID-19 vaccination have had significant attention in the global media and surveillance schemes [38]. This has led to a surge of research in response to public concern. The Nurses’ Health Study of pre-menopausal female health professionals compared baseline menstrual parameters collected in 2011–2016 with those collected in 2021 from 3858 participants, 3527 of whom were vaccinated against COVID-19 [2]. Pre- to post-COVID-19 analysis revealed that vaccinated women had a higher risk of increased cycle length than unvaccinated women (odds ratio: 1.48; 95% confidence interval: 1.00–2.19), after adjusting for sociodemographic and health behaviours. The ability to follow-up participants in this longitudinal study is a major strength and the association between menstrual disturbance and vaccination was only detected in the first 6 months. Another study used prospectively tracked menstrual cycle data from a menstrual app to limit recall bias [39,40]. Researchers analysed menstrual cycle length and menstrual duration from users in the US with regular menstrual cycles who were not using hormonal contraception. They compared pre-vaccination cycles with vaccination cycles in those who were vaccinated (*n*=14,936) and a similar 4-month window in unvaccinated users (*n*=4686). COVID-19 vaccination changed cycle length by <1 day and had no significant effect on menstrual duration. Participants who had both doses of the COVID-19 vaccine in the same cycle were most affected. Cycle length returned to normal within two cycles post vaccination. Participants all had regular cycles and were not using exogenous hormones, limiting confounders. However, the study population is not representative of the wider population. The Apple women’s health study also used menstrual tracking data from 9652 pre-menopausal women (8486 vaccinated; 1166 unvaccinated) who were not using exogenous hormones but did not limit to those with regular cycles and included those with pre-existing gynaecological disorders [41]. Participants received an mRNA vaccine (Pfizer-BioNTech or Moderna) or the J&J viral vector vaccine. This study revealed a small increase in menstrual cycle length in vaccination cycles, when compared with pre-vaccination cycles (first dose: 0.5 days, second dose: 0.39 days). These changes resolved in subsequent cycles and were associated with vaccine administration in the first half of the cycle. Of note, there were no differences in mean cycle length between vaccinated and unvaccinated participants.

Beyond menstrual cycle length, other studies have reported various changes in regularity, duration and volume of menstruation. Between May and August 2021, 51074 women aged 18–30 years were registered in the Norwegian National Population Registry [42]. A total of 8576 female participants consented to complete electronic questionnaires about menstrual disturbance before and in the first 6 weeks after COVID-19 vaccination. These methods were designed to limit selection bias and response rate was 67%. Of 5765 female respondents, 3972 menstruated regularly and had received 1 or 2 doses of COVID-19 vaccination. Prior to vaccination, 36.7% of participants reported at least one change from normal, highlighting the high level of variation in usual menstrual cycles. The relative risk of heavier bleeding than usual was 1.90 (95% CI: 1.69–2.13) after the first vaccine dose and 1.84 (95% CI 1.66–2.03) after the second dose and this did not differ with vaccine brand, hormone use or pre-existing gynaecological conditions.

Another prospective study of 79 participants recruited via social media in the UK examined menstrual volume, frequency and duration using menstrual diaries over three consecutive menstrual cycles, at least one of which included COVID-19 vaccination [43]. The subsequent menstrual episode following vaccination occurred a mean of 2.3 days late after dose 1 and 1.3 days late after dose 2 in spontaneously cycling individuals. This delay was not observed in those taking hormonal preparations, although the study was not sufficiently powered to detect small changes, and the brand of vaccination did not impact results. There were no significant differences in menstrual flow volume reported by participants after COVID-19 vaccination.

A gender-diverse sample of 39129 individuals who had received COVID-19 vaccination and had not had acute COVID-19 in the United States completed a web-based survey on menstruation between April and June 2021 in response to widespread media attention [44]. Following COVID-19 vaccination, 42% of people with regular menstrual cycles bled more heavily than usual, while 44% reported no change after being vaccinated. Among respondents who typically do not menstruate, 71% of people on long-acting reversible contraceptives, 39% of people on gender-affirming hormones, and 66% of postmenopausal people reported breakthrough bleeding. These data are important for patient counselling and for the planning of clinical services in current and future pandemics. The major limitation of this study is the potential for selection and recall bias and the lack of a comparator group of unvaccinated individuals.

The mean number of heavy menstrual bleeding days (fewer, no change or more) and changes in bleeding quantity (less, no change or more) was assessed in 9555 regularly cycling individuals not using exogenous hormones (7401 vaccinated and 2154 unvaccinated) who prospectively collected data using a menstrual app over at least 4 cycles [45]. There were no differences in number of heavy bleeding days between vaccinated and unvaccinated participants in first-dose, second-dose or post-exposure menses. Approximately 34.5% of unvaccinated participants reported ‘more’ bleeding quantity compared to 38.4% of vaccinated participants (adjusted difference 4.0%, 99.2% CI: 0.7–7.2%). These differences resolved in the subsequent cycle.

A UK web-based questionnaire collected data on COVID-19 and menstrual parameters in March–June 2021, prior to widespread UK media attention to menstrual disturbances following COVID-19 vaccination [33]. Of almost 5000 women who had at least one vaccination, 82% reported no changes to their periods. Reported changes were diverse and more likely in those who smoked, who had previously had acute COVID-19 and were less likely if using estradiol containing contraceptives. The timing of this study provided a unique opportunity to compare menstrual parameters in those (i) who were vaccinated but had never had acute COVID-19 (*n*=3653, vaccination group), (ii) were unvaccinated and had acute COVID-19 (*n*=1802, acute COVID group) and (iii) who were unvaccinated and who never had acute COVID-19 (*n*=5788, controls). Menstrual symptoms were not significantly different in the COVID-19 vaccination group when compared with controls, highlighting that menstrual changes are common in the general population. Of note, in contrast to COVID-19 vaccination, those who had acute COVID-19 showed an increased risk of reporting frequent cycles (<24 days), prolonged periods (>8 days), heavier period flow and inter-menstrual bleeding.

Taken together, these studies provide invaluable data on menstruation and vaccination. Despite variation in research methodologies and populations studied, these studies have consistently shown that the menstrual changes reported were not significantly affected by vaccine brand [33,43,44], in line with reports made on the UK Yellow Card surveillance scheme [38] and consistent with changes reported with vaccination against other pathogens [34,35]. These historical and current data are consistent with menstrual disturbance being a consequence of the immune response to vaccination, rather than individual components of the COVID-19 vaccination and potential immune pathways have been reviewed recently [46]. Furthering our understanding of these proposed mechanisms is critical to inform evidence-based discussions with those experiencing vaccine hesitancy. Studies to date are also consistent in their findings that any menstrual disturbance related to vaccination is transient and may be limited to the vaccination cycle or completely resolve by 6–9 months [34,39,45,47], providing further reassurance and information for those who menstruate. The current evidence indicates that a significant proportion of women will report menstrual disturbance following vaccination and it is useful for clinicians to be able to provide information about the likelihood of an individual experiencing such symptoms. There is some evidence that those using estrogen-containing contraceptives are protected from menstrual disturbance post vaccination [33,43] and that smoking increases the risk [33]. The effect of the timing of vaccination within the menstrual cycle remains unclear [41,43]. The association between pre-existing gynaecological conditions and self-reported changes is variable across studies [33,44], consistent with there being no strong association. Therefore, clinicians should elicit a medical history to determine risk factors for possible menstrual effects following COVID-19 vaccination and tailor their counselling to individual women. Providing reassurance about the transient nature of such effects should be balanced with the advice to attend if they are severe, lasting more than 6 months or involve ‘red flag’ symptoms such as inter-menstrual bleeding, post-coital bleeding, or post-menopausal bleeding, in line with current clinical guidance [18,48].

## The association between long COVID and AUB

The experience of sustained post COVID-19 infection sequalae is known as long COVID or long haul COVID. A recent World Health Organisation-led Delphi process reached a consensus that post-COVID-19 condition occurs in individuals with a history of probable or confirmed SARS-CoV-2 infection, usually 3 months from the onset, with symptoms that last for at least 2 months and cannot be explained by an alternative diagnosis [49]. A patient led survey of those experiencing long COVID symptoms revealed nine main symptom clusters: systemic, reproductive/genitourinary/endocrine, cardiovascular, musculoskeletal, immunologic/autoimmune, head eyes ear nose and throat, pulmonary, gastrointestinal and dermatologic [50]. Evidence suggests that long COVID affects twice as many women as men and, of those under 50, women were five times less likely to report feeling recovered than men of the same age [51]. Despite this, reproductive health conditions are significantly understudied in those with long COVID [7].

The patient led survey of long COVID symptoms described above included 1792 participants currently experiencing menstruation. Menstrual/period issues were reported by 33.8%, including abnormally irregular periods (26%) and abnormally heavy periods/clotting (19.7%) [50]. Due to a focus on all symptomatology, rather than menstrual symptoms per se, the use of exogenous hormones was not reported. A Spanish online survey of 17,455 people who menstruate carried out March to July 2021 revealed menstrual disturbance during this time was common (39.4%) [52]. Those with suspected/diagnosed long COVID (*n*=748) had an increased risk of self-reported menstrual alterations when compared to those who had never had COVID-19 (OR: 1.34, 95% CI: 1.15–1.57, *P*<0.001) and the risk was higher than those with acute COVID-19 who recovered. This study adjusted for known confounders, such as pre-existing gynaecological conditions and hormone use but details of the type of menstrual alterations was not reported. These initial data imply that those experiencing long COVID have an increased risk of reporting menstrual disturbance. To date, large studies that both detail the specific menstrual symptoms experienced by those with long COVID and consider potential confounders have yet to be carried out.

## Potential mechanisms for AUB associated with COVID-19

Taken together, the studies discussed above provide evidence of an association between acute COVID-19/long COVID and menstrual disturbance. However, methodological challenges (including self-reporting bias, small or non-representative sample sizes, cross-sectional data, and confounding factors) mean that it is currently unclear whether these associations represent causal relationships. There is some evidence that pandemic related stress did not result in significant population-level changes to ovulation or menstruation [53], consistent with a more specific impact of SARS-CoV-2 infection on menstruation. The acute menstrual disturbance described with COVID-19 illness/SARS-CoV-2 infection makes a non-structural cause for AUB more plausible than structural causes. In this section, we outline the physiology of menstruation before exploring the potential for abnormal uterine bleeding due to non-structural causes, specifically: coagulation disturbance (AUB-C), ovulatory dysfunction (AUB-O), disturbance of endometrial function (AUB-E) and iatrogenic causes (AUB-I) after SARS-CoV-2 infection.

## Physiology of menstruation

An understanding of menstrual physiology is essential in delineating the mechanisms that may lead to AUB in SARS-CoV-2. Menstruation is the breakdown and shedding of the luminal portion of the endometrium, which occurs in the absence of pregnancy. Endometrial function is tightly regulated by the hypothalamic–pituitary–ovarian axis. Pulsatile secretion of gonadotrophin-releasing hormone (GnRH) from the hypothalamus acts on the pituitary to release follicle stimulating hormone (FSH) and luteinising hormone (LH), which stimulate the development of ovarian follicles and the secretion of oestradiol and progesterone. Oestradiol is the dominant hormone in the proliferative or follicular phase. Progesterone is released from the corpus luteum after ovulation and is the principal hormone during the secretory or luteal phase. Their actions on the endometrium result in complex interactions between the endocrine, vascular and immune systems.

Progesterone and oestradiol levels decline sharply if implantation does not occur and the corpus luteum regresses during the late secretory stage. This results in local endometrial tissue inflammation with an influx of a host of inflammatory chemokines and cytokines and subsequent leukocytes [54–56]. This endometrial inflammation culminates in breakdown of the endometrium by matrix metalloproteinases (MMPs) and menstrual bleeding [57–59]. Endometrial tissue repair is then required to limit blood loss and maintain endometrial function in subsequent cycles. This endometrial repair occurs at the time of menstruation, with adjacent areas of endometrium undergoing breakdown and repair [60]. A transient, localised tissue hypoxia has been detected in the endometrium at menstruation [61,62] and was required for timely repair of the denuded endometrial surface [63]. A functional coagulation system is also required to limit menstrual blood loss, with the clotting cascade and platelet function important for achieving haemostasis post menstruation (Figure 2) [64,65].

## COVID-19 and potential AUB-C

Acute COVID-19 is associated with thrombosis, with the rates varying from 6 to 26% depending on the severity of the disease, age, race, hospitalisation status and prophylactic anticoagulant use [66,67]. Interestingly, thrombosis due to COVID-19 appears to have a different mechanism than that due to bacterial infection. The incidence of disseminated intravascular coagulation was only 3% in those with COVID-19, in comparison with rates of 20–70% in those with bacterial infections [67,68]. Hypercoagulable and hyperfibrinolytic states are predominant in bacterial infections, whereas hypercoagulable and hypofibrinolytic states with platelet activation are predominant in COVID-19 [67].

Women with HMB have also been reported to have disturbance of the fibrinolytic system. Those experiencing HMB have raised levels of tissue plasminogen activator activity in the endometrium during menstruation when compared to those with normal menstrual loss, consistent with an overactive fibrinolytic system [69]. This finding is supported by the therapeutic effectiveness of tranexamic acid, an antifibrinolytic medication that resulted in a 58% reduction in menstrual blood loss [70]. Hence, the systemic hypofibrinolytic effects observed in those with COVID-19 may explain some of the diversity of menstrual disturbance experienced, causing lighter bleeding or spotting.

Platelet adhesion and activation are likely to have a significant impact on menstrual blood loss, evidenced by the high prevalence of von Willebrand disease in those with HMB (13%) [71]. In contrast, those with COVID-19 have been found to have significantly elevated levels of von Willebrand factor antigen and activity, consistent with a pro-thrombotic profile [72]. Although the relationship between systemic and local endometrial coagulation is unlikely to be linear, the fact that the coagulation system is important for menstrual regulation and that there are significant perturbations with COVID-19 presents a potential mechanistic pathway for menstrual disturbance (Figure 2B), in particular amenorrhoea or lighter menstrual bleeding, in those with COVID-19 that merits further investigation.

## COVID-19 and potential AUB-O

SARS-CoV-2 infection and COVID-19 illness have the potential to affect the HPO axis with resulting changes to the menstrual cycle. Hypothalamic hypogonadism may occur in the presence of any severe illness, including COVID-19, and result in temporary amenorrhoea or infrequent menses (Figure 2B). This protective mechanism enables diversion of energy resources from reproduction to the immune response [73]. There is evidence for an altered immune response in those requiring hospitalisation for COVID-19 and persistent immune dysregulation [74]. Furthermore, immune cell dysregulation has been suggested as a potential cause of experiencing long-term symptoms of COVID-19 [74,75] highlighting the importance of prioritising the regulation of the adaptive immune response for COVID-19 recovery. Effects on the HPO axis may also explain why those experiencing long-term symptoms of Ebola infection (Post Ebola Syndrome; possibly analogous to long COVID) have also reported menstrual cessation or irregularity [76]. Current evidence suggests that the association between COVID-19 and menstrual cycle disturbances is driven by immunological effects caused by SARS-CoV-2, rather than disease-related stress alone. Of note, examination of ovulation and menstrual parameters using a mobile tracking app comparing March to September 2020 (during the pandemic) to March to September 2019 (pre-pandemic) revealed no population level changes and no correlation between reported stress levels and menstrual cycle abnormalities [53]. This suggests that pandemic-related stress did not significantly impact the HPO axis. The COVID-19 status of participants was unknown, but the number of positive cases is likely to be low as the study was completed early in the pandemic, and findings may be different in those with significant or prolonged symptoms of COVID-19.

SARS-CoV-2 uses the angiotensin-converting enzyme 2 (ACE2) and the cell protease type II transmembrane serine protease (TMPRSS2) to bind to the cell and for virus–cell fusion [13,14]. The *ACE2* gene is expressed on ovarian tissue at similar levels to that found in the lung [77]. A study examining tissue distribution of ACE2 noted that it was present in arterial and venous endothelial cells and arterial smooth muscle cells in all organs studied, although reproductive tissue was not included [78]. Co-expression of *ACE2* and *TMPRSS2* was detected using scRNA-seq in human ovarian cortex and medulla, and oocytes [14,79,80], indicating the potential for ovarian cells to be prone to infection. These studies did not record whether participants experienced symptoms of AUB. To examine ovarian function, ovarian sex hormone levels during the follicular phase were measured in 91 aged-matched women with and without COVID-19 and revealed no significant differences between the groups [27]. Another study of 78 patients with COVID-19 detected lower AMH levels and higher testosterone levels when compared to aged-matched controls [29]. Whether COVID-19 transiently affects ovarian sex hormone production and/or ovulation remains an active research question [81] and further investigation will reveal the contribution of ovulatory dysfunction to the presentation of AUB in those with acute and long COVID.

## COVID-19 and potential AUB-E

Alternatively, or additionally, there may be effects of SARS-CoV-2 infection on the local endometrial environment that impact menstruation. A study examining endometrial transcriptomic datasets (microarray or bulk RNA sequencing) from 112 participants at different stages of the menstrual cycle found that gene expression was medium for *TMPRSS2* and low for *ACE2* but that endometrial *ACE2* increased with age [82]. These findings led authors to conclude that endometrial tissue is at low risk of SARS-CoV-2 cell entry, but that susceptibility increases with age. A study of healthy human endometrium from 27 donors obtained across the menstrual cycle using single cell RNA sequencing also concluded that endometrial SARS-CoV-2 infection risk was low, with very few cells of any type exhibiting co-expression of *ACE2/neuropilin-1 (NRP1)* or *ACE2/NRP1/TMPRSS2* at any stage of the menstrual cycle [83]. Both these studies examined endometrium from women without acute COVID-19 and did not record whether participants experienced AUB but suggest that a direct impact of SARS-COV-2 infection on the endometrium is unlikely.

A more indirect effect on endometrial function is possible, as the endometrium is susceptible to systemic inflammation and responses, with influx of circulating immune cells contributing to menstrual physiology [84,85]. RNA sequencing of endometrium from six women hospitalised with moderate-to-severe COVID-19 and eight controls revealed differential endometrial gene expression [86]. Processes such as subcellular component movement, response to stimulus and pathways corresponding to viral response were up-regulated in the endometrium of those with COVID-19, while digestive and endocrine system and developmental processes and immune cell regulation pathways were down-regulated. These findings suggest that endometrial inflammation is altered in COVID-19, but the results of this study should be interpreted with caution. Of the control samples, six were from the proliferative phase, compared to only one of the COVID-19 samples. Authors state this was accounted for in analysis, but the heterogeneity of the samples is a major limitation of the study given the low numbers. This study was also unable to differentiate if the effects observed were unique to COVID-19 or due to any systemic viral infection.

The same group performed integrated *in silico* analysis of two datasets [87]. The first set from nasopharyngeal swabs from 231 women with confirmed COVID-19 (standardised at 7 days post symptom onset) and 30 women without. The second set was from secretory endometrium from women without endometrial pathology. This *in silico* model predicted that five genes important for embryo implantation were affected by COVID-19 (down-regulation of *COBL, GPX3* and *SOCS3*, and up-regulation of *DOCK2* and *SLC2A3*). The findings were validated in endometrial samples from women with and without COVID-19 (*n* = 3/group proliferative and *n* = 5/group secretory). Due to low numbers, there were no statistically significant changes. *COBL, GPX3* and *SOCS3* were lower in the early and late secretory phase in women with COVID-19, whereas *DOCK2* and *SLC2A3* were unchanged. The authors suggest that COVID-19 changes the transcriptomic landscape of endometrial receptivity genes and key processes such as immune system function, protection against oxidative damage and development. The findings require validation in a larger dataset but suggest that secretory endometrial physiology may be affected by COVID-19 and therefore contribute to menstrual disturbance.

To date there are no studies examining the impact of COVID-19 on the menstrual phase endometrial transcriptome. Aberrations in endometrial menstrual physiology are known to result in AUB-E, including effects on local inflammation, vascular function, hypoxia and tissue repair. The menstrual endometrium has many of the classic hallmarks of inflammation with an influx of inflammatory mediators and cells following progesterone withdrawal. There is evidence that this inflammatory response is excessive in those experiencing AUB. The pro-inflammatory cytokine tumour necrosis factor α (TNFα) was found to be higher in menstrual effluent from women with HMB compared with normal controls [88]. The role of the prostaglandin synthesis pathway was also found to be aberrant in women with objective HMB. Analysis of gene expression in endometrial biopsies from the secretory phase revealed significant elevation of *COX-2* mRNA in women with blood loss greater than 80 ml [89]. Increased levels of total prostaglandins were detected in the endometrium of women with HMB [90,91]. Increased signalling of Prostaglandin (PG) E_2_ through its EP2 and EP4 receptors has also been proposed due to elevated production of cyclic AMP in endometrium from women with HMB versus normal bleeding. This exaggerated inflammation may lead to increased and prolonged tissue damage at the time of menstruation and result in AUB. In support of the importance of a controlled endometrial inflammatory response at menstruation, non-steroidal anti-inflammatory medications taken regularly during menstruation are an effective treatment for HMB [92]. It is not yet known if COVID-19 results in excessive or altered endometrial tissue inflammation. A recent study of blood and lung tissue from COVID-19 patients, healthy controls and patients with other respiratory conditions revealed that a subset of monocytes can be infected with SARS-CoV-2 via the CD16 receptor, leading to inflammatory cell death and release of inflammatory mediators [93]. Lung tissue resident macrophages also had activated inflammasomes in COVID-19 patients. Given the key role of monocytes in endometrial breakdown and repair [84], it is possible that COVID-19 alteration of inflammatory cell phenotype could increase local endometrial inflammation and result in AUB (Figure 2B).

There is mounting evidence that endothelial cell dysfunction is a key mechanism in COVID-19 pathogenesis [94,95]. Histopathological assessments have demonstrated that COVID-19 is a microvascular and endothelial disease [95,96] and found that the ACE2 receptor was present in arterial and venous endothelial cells and arterial smooth muscle cells in all organs studied [78]. COVID-19-related endothelial dysfunction in the form of aberrant vascular tone, excessive inflammation, cytokine storm, oxidative stress, endothelial mesenchymal transition, mitochondrial dysfunction, virus-induced senescence and coagulopathy has been reported [97,98]. As well as the endothelial dysfunction associated with acute lung injury [99], COVID-19 has been shown to be a risk factor for more systemic endothelial dysfunction, for example, increasing the risk of myocardial infarction and ischaemic stroke [100]. The evidence for end organ endothelial cell dysfunction resulting in pathology raises the possibility that endometrial vascular function could be perturbed following SARS-CoV-2 infection, either directly or indirectly, resulting in menstrual disturbance. To our knowledge, there are no currently published studies comparing the endometrial endothelial transcriptome or histological features in those with and without COVID-19. Until this evidence is available, we hypothesise that vascular/endothelial dysfunction caused by COVID-19 has the potential to affect the conditioning of the spiral arterioles throughout the menstrual cycle, resulting in irregular or unscheduled menstrual bleeding, and/or could result in ineffective vasoconstriction during menstruation and HMB (Figure 2B). The specialised endometrial spiral arterioles are critical for bleeding limitation during menstruation. Following progesterone withdrawal in the late secretory phase, these spiral arterioles vasoconstrict to limit menstrual blood loss [101]. Prostaglandin (PG) F_2α_ and endothelin-1 (ET-1) increase in the endometrium at the time of menses and have known vasoconstrictive properties [102,103]. Women with objectively measured HMB were found to have a significantly decreased PGF_2α_/PGE_2_ ratio [90] and decreased endometrial expression of the potent vasoconstrictor ET-1 [103]. Additionally, or alternatively, a lack of spiral arteriole maturation may also contribute to inefficient spiral arteriole vasoconstriction at menstruation. Endometrial vessel wall circumference and focal discontinuities were noted to be larger in women with HMB than in normal controls [104] and had significantly reduced vascular smooth muscle cell proliferation [105] and smooth muscle myosin heavy chain expression [106].

A lack of spiral arteriole vasoconstriction during menstruation may not only increase menstrual flow but also limit or delay repair of the denuded endometrial surface required for cessation of menstrual bleeding. A transient physiological hypoxia is present in the upper endometrial zones at menstruation [61,62] and has been shown to drive endometrial menstrual repair [63]. Endometrial hypoxia inducible factor (HIF)-1α was present exclusively during the peri-menstrual phase and women with HMB were found to have lower endometrial HIF-1α protein and its downstream targets involved in angiogenesis and tissue repair [63,88]. This physiological hypoxic response appears tightly regulated to coordinate the tissue repair process necessary to limit menstrual bleeding. Acute COVID-19 can result in very low oxygen saturation, with 20–40% of people experiencing ‘silent’ or asymptomatic hypoxia [107,108]. It is likely that hypoxaemia affects uterine function, as evidenced by features of fetal hypoxic-ischaemic injury and placental defects in cases of stillbirth and neonatal death to mothers with SARS-CoV-2 infection during pregnancy [109]. Alteration of endometrial hypoxic pathways, with potential activation of HIF-1 outside the physiological peri-menstrual period, could theoretically alter endometrial angiogenesis/metabolism and lead to abnormal uterine bleeding (Figure 2B). Consistent with this theory is the finding that metabolism was one of the main functions dysregulated in endometrial tissue from six patients with COVID-19, although sample size was limited in this pilot study [86].

## COVID-19 and potential AUB-I

Iatrogenic causes of AUB include use of anti-coagulant medications, gonadal steroids (e.g. estrogens, progestins, androgens) and nonsteroidal pharmaceuticals that contribute to ovulatory disorders (e.g. phenothiazines and tricyclic antidepressants) [15]. It is important to remain cognisant that treatments for COVID-19 may have off target effects that result in menstrual disturbance. For example, some of the common anti-viral medications used to treat early COVID-19 symptoms contain cytochrome P450 inhibitors (e.g. ritonavir). Concomitant use of hormonal contraception and drugs that inhibit cytochrome P450 has the potential to result in increased exposure to contraceptive hormones and increased side effects. This is a potential mechanism for reports of menstrual disturbance in those using hormonal contraception and who received treatment with anti-viral medications (Figure 2B), although these numbers are likely to be small.

In patients hospitalised with COVID-19, low-dose corticosteroids in the form of dexamethasone have been shown to reduce mortality in those requiring oxygen or ventilatory support [110]. Endogenous glucocorticoids have been shown to inhibit endometrial angiogenesis [111] and regulation of endometrial blood vessel function is required to limit endometrial bleeding and menstrual blood loss [17,112]. A recent response-adaptive randomised placebo-controlled dose-finding parallel group trial revealed that low dose dexamethasone taken in the luteal phase of the menstrual cycle reduced menstrual blood loss [113]. Exposure to exogenous dexamethasone at other phases of the cycle may affect endometrial vasculature and represent a potential mechanism of reported menstrual disturbance in those with severe acute COVID-19 (Figure 2B).

Given scientific research on long COVID is in its infancy, many of those suffering with longer term symptoms of COVID-19 are turning to off-label treatment options. There are many anecdotal reports of hyperbaric oxygen therapy (HBOT) improving long COVID symptoms. One study found statistically significant improvements in fatigue and cognitive scoring in 10 participants with long COVID after 10 sessions of HBOT to 2.4 atmospheres over 12 days [114]. This study was small and had no control group. A small (*n*=73) randomised, placebo-controlled, double-blind trial has also shown benefits of daily HBOT [115] and a phase II clinical trial is in progress [116]. During HBOT, the arterial O_2_ tension typically exceeds 1500 mmHg, and levels of 200–400 mmHg occur in tissues [117], consistent with it having an impact on endometrial tissue oxygen levels. Mice placed in hyperoxic conditions following progesterone withdrawal during simulated menstruation displayed less endometrial hypoxia and delayed endometrial repair [63]. The effects of intermittent HBOT on endometrial function remain undetermined, but prevention of physiological endometrial hypoxia has the potential to result in HMB [63]. Alternatively, it has been proposed that intermittent exposure to hyperoxic conditions and the relative changes in oxygen availability paradoxically stabilises HIF protein [117,118] and promotes angiogenesis. Given the presence and role of endometrial hypoxia in driving repair at menstruation, it is important to determine the impact of HBOT on menstrual symptoms and physiology. In addition, these studies highlight the importance of COVID-19 treatments as confounders when examining the impact of COVID-19 on menstruation.

These hypothesised non-structural mechanisms that may underpin abnormal uterine bleeding with COVID-19 (AUB C/O/E/I, Figure 2) require investigation to ensure specific, evidence-based management. Although currently speculative, the FIGO AUB system 2 [15] provides a useful framework to design future research in this area, combining our knowledge of COVID-19 effects on other systems.

## The impact of the menstrual cycle on COVID-19

**Figure 3:**
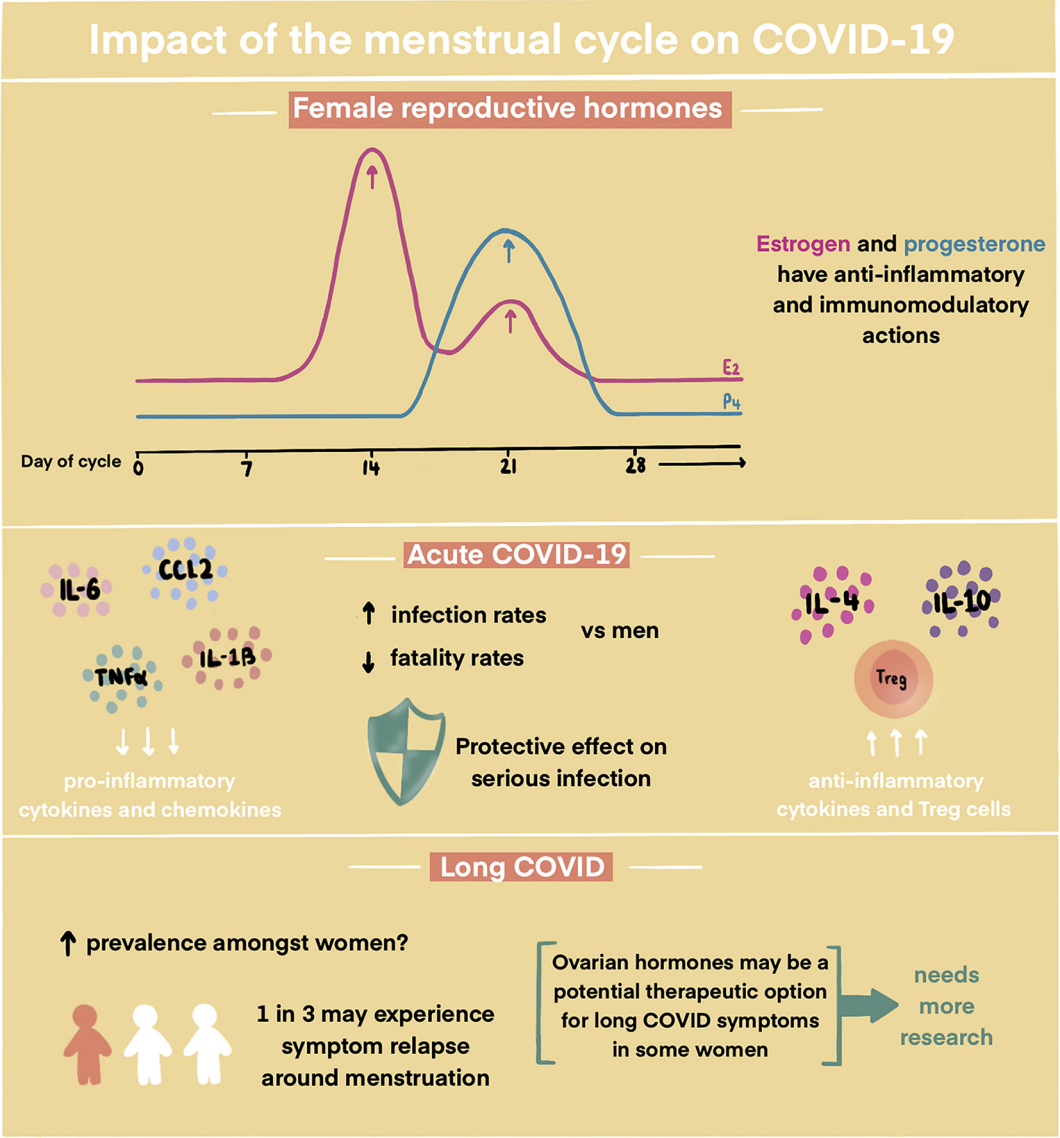
The impact of the menstrual cycle on COVID-19 The potential role of ovarian hormones, estrogen (E_2_) and progesterone (P_4_) on the development and symptomatology of acute COVID-19 and long COVID (IL, interleukin; CCL2, chemokine ligand 2; TNF, tumour necrosis factor; Treg: regulatory T cells).

Early in the COVID-19 pandemic it became apparent that there were differences in the response to SARS-CoV-2 infection in males and females. Examination of COVID-19 reported deaths from 16 countries in a 6-week period in 2020 revealed mortality rates from COVID-19 were 77% higher in men than in women (IRR = 1.77, 95%CI = 1.74, 1.79) [119]. This is likely to be due to a combination of gender-specific behaviours/activities/roles and biological sex differences. A Swedish cohort of post-menopausal women who tested positive for SARS-CoV-2 was divided into three categories for analysis: [1] decreased systemic estrogen levels [2], increased estrogen levels and [3] controls [120]. This revealed an absolute risk of death of 4.6% for the control group vs 10.1% and 2.1%, for the decreased and increased estrogen groups, respectively. This was despite adjustment for age, annual disposable income, highest level of education and the weighted Charlson Comorbidity Index. Further evidence for the reproductive hormones altering COVID-19 response comes from analysis of electronic health records in a large, international COVID-19 cohort, grouped by age. Pre-pubertal males and females had the same risk of infection and fatality rate, adult pre-menopausal women had a significantly higher rate of infection than men of the same age but lower fatality rate and post-menopausal women had a similar severe infection rate as men in the same age category [121]. Subanalysis of women over 50 using estradiol containing hormone replacement therapy revealed a 50% reduction in fatality rate [121]. In addition, a population based matched cohort study of female users of the COVID Symptom Study application in the UK revealed menopausal women had significantly higher rates of predicted COVID-19 and combined oral contraceptive pill (COCP) users had lower rates of predicted COVID-19 and reduced hospital attendance (*P*=0.023). Taken together, these studies are consistent with a protective effect of the female reproductive hormones on serious SARS-CoV-2 infection and mortality (Figure 3).

One mechanism underpinning these sex differences in acute COVID-19 outcomes may be the decreased innate immune inflammatory response and enhanced immune tolerance and antibody production in the presence of higher levels of estradiol and progesterone [6] (Figure 3). It has long been known that females develop a heightened immune response to viruses than males [122]. Estradiol and progesterone have known anti-inflammatory and immunomodulatory actions [6,123–126], including suppression of the production of proinflammatory cytokines and chemokines (e.g. interleukin 6, interleukin 1β, TNF-α and CCL2), stimulation of CD4+ T-helper cell production of anti-inflammatory cytokines, and increase Th2-type anti-inflammatory responses (Figure 3). Estradiol and progesterone can also enhance the expansion of regulatory T cells to promote immune tolerance. Collectively, these alterations may help to prevent or limit the cytokine storm associated with severe COVID-19.

In addition to its impact on acute COVID-19, there is mounting evidence that the menstrual cycle may affect the development and symptomatology of long COVID (Figure 3). A UK study of 327 hospitalised patients found that women under 50 were five times less likely to report feeling recovered at least 90 days following symptom onset when compared with men of the same age [51], suggesting the ovarian sex hormones may play a role in the pathogenesis of long COVID [127]. An online survey of 3762 people from 56 countries experiencing symptoms of COVID-19 lasting over 28 days included 2348 women <50 years old. More than a third of menstruating participants experienced relapses of their long COVID symptoms during or before menstruation [50]. Many patients with long COVID describe symptoms consistent with postural orthostatic tachycardia syndrome (POTS). In a study carried out prior to the COVID-19 pandemic, women with POTS were shown to have lower cardiac output and stroke volume and greater total peripheral resistance in the early proliferative phase of the cycle than mid secretory phase [128]. No such variation in parameters was observed in normal controls across the menstrual cycle. Hence, ovarian sex hormones may provide a therapeutic option for long COVID symptoms in some women and their safety profile and effectiveness should be investigated further (Figure 3). Treatments targeting these ovarian hormones have been investigated as a treatment for acute COVID-19. A phase 2 randomised, double-blinded, placebo-controlled, multicentre trial evaluated the efficacy and safety of raloxifene treatment (a second-generation estrogen receptor modulator) for patients with mild-to-moderate COVID-19 [130]. The proportion of participants with undetectable SARS-CoV-2 after 7 days of treatment with raloxifene 60 mg and 120 mg was lower compared to placebo, RD = 0.37 (95% CI: 0.09 to 0.59) and RD = 0.22 (95% CI: −0.03 to −0-.45), respectively. There was no effect on requirement for supplemental oxygen or mechanical ventilation. Another phase 2, randomised, double-blind, placebo-controlled trial investigated the efficacy, safety and tolerability of estetrol (E4) versus placebo in hospitalised patients with SARS-CoV-2 [131]. The primary endpoint of improvement in recovery at day 28 was not altered between groups.

## Conclusions

A lack of robust menstrual data during the COVID-19 pandemic made it difficult for the public and health professionals to determine the impact of acute SARS-CoV-2 infection, long COVID or COVID-19 vaccination on menstruation. We have reviewed available evidence and highlight that many studies from this time are limited by lack of standardised data collection, recall bias, variable sample sizes and confounders. Despite these limitations, collectively these studies suggest that SARS-CoV-2 infection (acute COVID-19 and long COVID) is associated with reported changes in menstrual bleeding parameters. Accurate information on the relationship between COVID-19 vaccination and menstrual disturbance is essential to avoid misinformation leading to vaccine hesitancy. Studies reviewed here report that some women may experience a small increase in cycle length or a change in bleeding pattern in the cycles following vaccination, but that this impact is transient and not specific to one brand of vaccine.

In addition, this review discusses potential mechanisms involved in COVID-19 related menstrual disturbance. Combining our understanding of AUB pathways with research on the pathogenesis of COVID-19 in non-reproductive systems highlights a range of potential mechanisms that require further investigation. COVID-19 may disrupt menstrual regulation due to an impact on the HPO axis or direct effects on immune function at level of the endometrium. We acknowledge that there is limited data available to confirm these proposed mechanisms and this presents an important area for further targeted study.

The number of people reporting symptoms of long COVID is increasing worldwide and there is growing interest to determine its impact across all body systems. Evidence presented here highlights that the menstrual cycle can impact long COVID symptomatology. This is an area which deserves further exploration to increase our understanding and develop better management strategies to improve quality of life.

The reporting of menstrual disturbance from COVID-19 generated public interest and concern that resulted in vaccine hesitancy. These concerns were exacerbated by a lack of clear explanations from the scientific and medical community due to a lack of data collection. Moving forward, data and research on COVID-19 and menstruation will inform policy and will be invaluable for future vaccine development and understanding post-viral syndromes. Menstrual data must be considered as an important health parameter and should be included in future studies of long COVID, other viruses and vaccination trials. This will ensure that women and people who menstruate can remain informed about the expected impacts of viral infection on menstrual bleeding and that we develop our understanding of the relationship between menstruation and health outcomes.

## Data Availability

Data sharing is not applicable to this review.

## Open Access

Open access for this article was enabled by the participation of The University of Edinburgh in an all-inclusive *Read & Publish* agreement with Portland Press and the Biochemical Society under a transformative agreement with JISC.

## Abbreviations

ACE2: angiotensin-converting enzyme 2
AMH: anti-Müllerian hormone
AUB: abnormal uterine bleeding
AUB-C: abnormal uterine bleeding due to coagulopathies
AUB-E: abnormal uterine bleeding due to primary endometrial disorders
AUB-I: abnormal uterine bleeding of iatrogenic origin
AUB-O: abnormal uterine bleeding due to ovulatory disorders
COBL: cordon-bleu WH2 repeat protein
COEIN: coagulopathies, ovulatory disorders, endometrial, iatrogenic and not otherwise classified
COVID-19: coronavirus disease 2019
DOCK2: dedicator of cytokinesis 2
EP2/EP4: prostaglandin E2 receptors
ET-1: endothelin-1
FIGO: the International Federation of Gynecology and Obstetrics
GnRH: gonadotropin hormone-releasing hormone
GPX3: glutathione peroxidase 3
HBOT: hyperbaric oxygen therapy
HIF: hypoxia-inducible factor
HMB: heavy menstrual bleeding
HPO: hypothalamic–pituitary–ovarian
HPV: human papillomavirus
LH: lutenizing hormone
MMP: matrix metalloproteinase
mRNA: messenger ribonucleic acid
NRP1: neuropilin-1
PALM: polyp, adenomyosis, leiomyoma, malignancy
PG: prostaglandin
POTS: postural orthostatic tachycardia syndrome
SARS-CoV-2: severe acute respiratory syndrome coronavirus 2
scRNA-seq: single-cell ribonucleic acid sequencing
SLC2A3: solute carrier family 2 member 3
SOCS3: suppressor f cytokine signalling 3
TMPRSS2: transmembrane serine protease type II
TNFα: tumour necrosis factor α
US: United States
